# The Distinctive Regulation of Cyanobacterial Glutamine Synthetase

**DOI:** 10.3390/life8040052

**Published:** 2018-10-27

**Authors:** Paul Bolay, M. Isabel Muro-Pastor, Francisco J. Florencio, Stephan Klähn

**Affiliations:** 1 Helmholtz Centre for Environmental Research, Department of Solar Materials, Permoserstrasse 15, D-04318 Leipzig, Germany; paul.bolay@ufz.de; 2 Instituto de Bioquímica Vegetal y Fotosíntesis, CSIC-Universidad de Sevilla, Américo Vespucio 49, E-41092 Seville, Spain; imuro@ibvf.csic.es (M.I.M.-P.); floren@us.es (F.J.F.)

**Keywords:** nitrogen assimilation, cyanobacteria, glutamine synthetase inactivating factors, glutamine riboswitches, non-coding RNAs

## Abstract

Glutamine synthetase (GS) features prominently in bacterial nitrogen assimilation as it catalyzes the entry of bioavailable nitrogen in form of ammonium into cellular metabolism. The classic example, the comprehensively characterized GS of enterobacteria, is subject to exquisite regulation at multiple levels, among them gene expression regulation to control GS abundance, as well as feedback inhibition and covalent modifications to control enzyme activity. Intriguingly, the GS of the ecologically important clade of cyanobacteria features fundamentally different regulatory systems to those of most prokaryotes. These include the interaction with small proteins, the so-called inactivating factors (IFs) that inhibit GS linearly with their abundance. In addition to this protein interaction-based regulation of GS activity, cyanobacteria use alternative elements to control the synthesis of GS and IFs at the transcriptional level. Moreover, cyanobacteria evolved unique RNA-based regulatory mechanisms such as glutamine riboswitches to tightly tune IF abundance. In this review, we aim to outline the current knowledge on the distinctive features of the cyanobacterial GS encompassing the overall control of its activity, sensing the nitrogen status, transcriptional and post-transcriptional regulation, as well as strain-specific differences.

## 1. Introduction

Nitrogen (N) is an essential element to all life on earth as it is a significant fraction of crucial biomolecules such as amino acids, nucleic acids and a myriad of derivatives. Despite being the most common pure element in the atmosphere, molecular dinitrogen (N_2_) can only be utilized by few archaea and bacteria that possess N_2_-fixing nitrogenase (EC 1.18.6.1). This enzyme reduces the inert triple bond of N_2_ by providing enormous reducing power to yield bioavailable, dissolved inorganic N in the form of ammonium, which can be taken up and assimilated into organic forms by most microorganisms [1]. Thus bioavailable N species mostly remain scarce and are subject to strong fluctuations in natural habitats rendering N availability a key environmental factor for growth [2,3]. Therefore, it is not surprising that most microorganisms frugally husband their N pools and strongly regulate uptake and assimilation machineries in response to environmental changes.

### 1.1. GS Catalyzes a Core Reaction of N Assimilation in Bacteria

**Figure 1:**
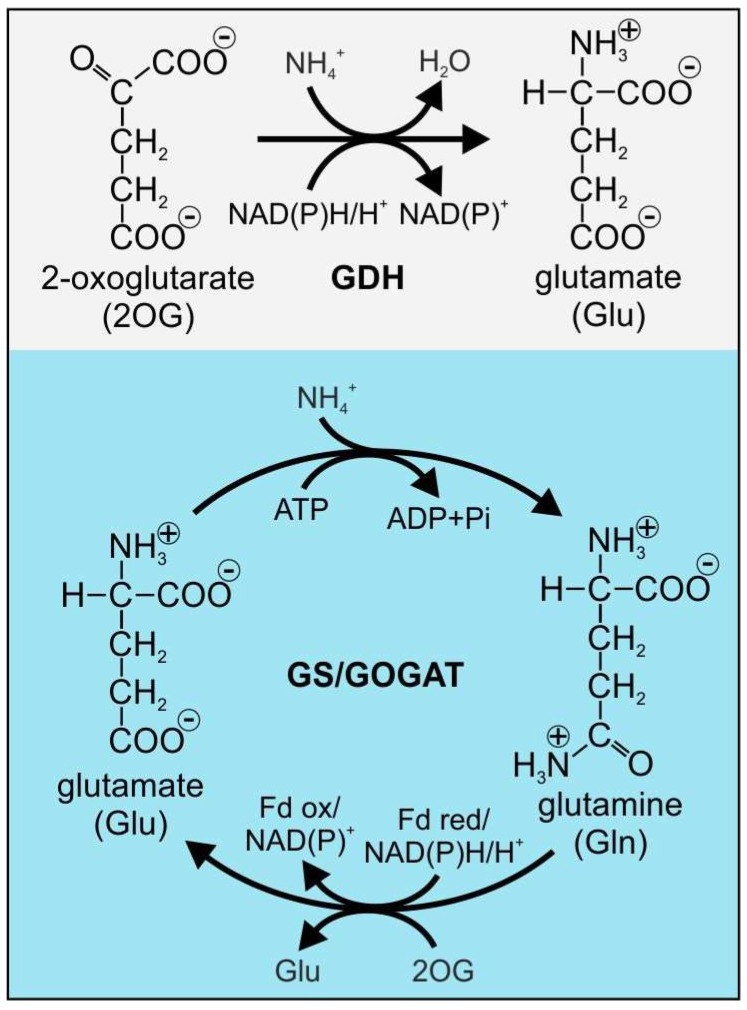
Ammonium assimilation reactions catalyzed by the glutamate dehydrogenase (GDH) and the glutamine synthetase/glutamate synthase (GS/GOGAT) cycle.

In *E. coli* and most other Gram-negatives, two primary pathways of ammonia assimilation have been described [4,5] (Figure 1). The initially discovered NAD(P)H/H^+^ glutamate dehydrogenase (GDH, EC 1.4.1.3) catalyzes the reductive, NAD(P)H/H^+^-dependent amination of 2-oxoglutarate (2OG) to yield glutamate and NAD(P)^+^, and was regarded as the main enzyme of ammonium assimilation in bacteria for a certain period [6]. Nevertheless, in 1970, Tempest and co-workers established another main N assimilatory system in bacteria, which comprises the subsequent action of the enzymes glutamine synthetase (GS; glutamate-ammonia ligase, EC 6.3.1.2) and glutamate synthase (formerly glutamine:2-oxoglutarate-aminotransferase (GOGAT), EC 1.4.1.14 (NAD(P)H^+^-dependent), and EC 1.4.7.1 (ferredoxin-dependent)). In the GS/GOGAT cycle GS facilitates the first step of N assimilation, the conversion of inorganic into organic N by incorporating ammonium into glutamate and yielding glutamine (Figure 1). Subsequently, with either NADPH/H^+^ or ferredoxin as reducing equivalents, GOGAT transfers the amine residue of glutamine to 2OG and forms two molecules of glutamate, which again serve as GS substrate [7,8]. Due to a greater affinity to ammonium and the dependence on ATP, the GS/GOGAT cycle is crucial for balancing glutamine pools and N-assimilation in enterobacteria and most Gram-negatives, i.e., during combined low ammonium availability and energy excess. In contrast, the GDH pathway is employed for glutamate synthesis upon energy limitation and ammonium profusion since the catalyzed reaction does not require ATP, while the enzyme features relatively high K_m_ values for ammonium [5].

The family of GSs constitutes one of the oldest existing and operational gene families [9]. GSs are found in all living organisms and are split into at least three miscellaneous types (GSI, GSII, and GSIII), which differ in subunit stoichiometry and molecular weight [10,11,12,13]. Interestingly, studies which revealed the wide dispersion of GS aroused the suspicion that the GS types evolved ahead of the divergence of eukaryotes and prokaryotes by early gene duplication [9,11,14,15]. While GSII represents the generic eukaryotic enzyme which, however, also occurs in representatives of the *Franciaceae*, *Rhizobiaceae* (both plant symbionts), and *Streptomycetaceae* families [16,17,18], GSI is restricted to bacteria and archaea [11]. The third GS type, GSIII is generally found in a myriad of bacteria but several eukaryotic representatives (e.g., members of fungi, viridiplantae, amoebozoa, heterokonts, and haptophyta) as well as archaea appear to also bear GSIII-encoding genes (see Pfam entry PF12437).

### 1.2. GS Regulation Is Diverse among Distinct Organisms

GSI (hereafter referred to as GS) consists of two superimposed hexameric rings, which are arranged in a centrosymmetric structure. The GS of enterobacteria is one of the best investigated and most comprehensively regulated enzymes known and represents the prevailing paradigm of bacterial GS regulation, which is addressed in most scientific textbooks. Enterobacterial GS is subject to feedback inhibition by several molecules of N and energy metabolism, which include among others tryptophan, histidine, carbamoyl phosphate, CTP, AMP, glucosamine-6-phosphate, NAD^+^, serine, and alanine, as well as glycine. These intermediates orchestrate a gradual inhibition of GS that mounts depending on the number of bound inhibitors, which is designated *cumulative feedback inhibition* [19,20,21]. The amino acids serine, alanine, and glycine were shown to interact with the glutamine binding site [22] and AMP appears to sequester the ATP binding cleft [23] while the remaining metabolites occupy distinct allosteric sites at the enzyme [20].

**Figure 2:**
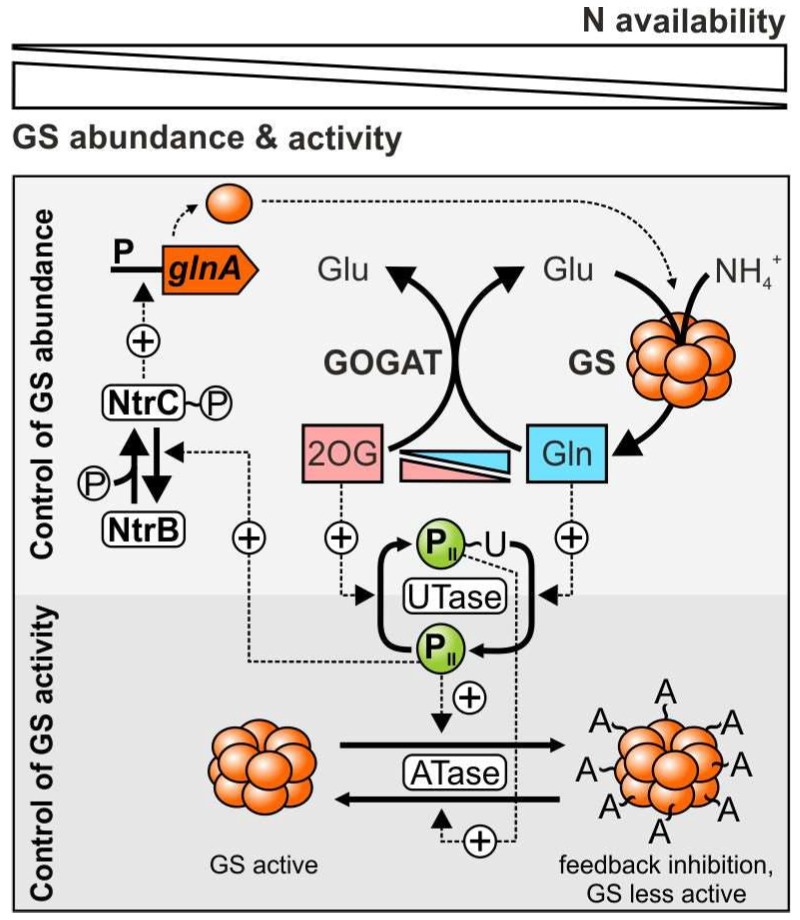
Simplified overview of N-dependent glutamine synthetase (GS) regulation in *E. coli*: GSI consists of two superimposed hexameric rings, which are arranged in a centrosymmetric structure. The bidirectional uridylyltransferase (UTase) senses both glutamine (Gln) and 2-oxoglutarate (2OG) and modifies the P_II_ signal transducer accordingly. Unmodified P_II_ promotes adenylylation of GS via the bidirectional adenylyltransferase (ATase), which enhances the sensitivity of the enzyme for feedback inhibition. Accordingly, modified P_II_ facilitates deadenylylation of GS, rendering the enzyme fully active. Moreover, by impacting the bidirectional kinase/phosphatase activity of the sensor kinase NtrB, P_II_ also regulates the NtrC-NtrB two-component system, which controls transcription of the GS encoding *glnA* gene. Under N excess, unmodified P_II_ promotes NtrB-dependent dephosphorylation of the response regulator NtrC, which prevents activation of *glnA* expression [38,39].

In addition, the enterobacterial GS is subject to posttranslational regulation by reversible adenylylation of specific tyrosine residues in each of the 12 enzyme subunits, which in turn raises the sensitivity of GS for feedback inhibition. The corresponding regulatory circuits, which govern the modification state of GS, are complex and involve two connected cycles of reversible protein nucleotidylation (Figure 2). In the first cycle, the ubiquitous signal transducing protein P_II_ switches between uridylylated and unmodified states [24,25], while in the second cycle GS changes between adenylylated and unmodified forms [26,27]. Uridylylation of P_II_ is catalyzed by the bifunctional uridylyltransferase (UTase) and depends on 2OG [24,25,28,29]. On the contrary, removal of the uridylyl moiety of P_II_ by UTase requires glutamine [30]. In an analogical process, adenylylation of GS by the bifunctional adenylyltransferase (ATase) is a glutamine-dependent reaction that is opposed by the 2OG-dependent deadenylylation of GS [26,31,32,33,34].

In general, sensing of the cellular C/N status is of utmost importance for regulating N uptake and assimilation to ensure the maintenance of metabolic homeostasis. Since 2OG provides the carbon skeleton for the amidation reaction in the GS/GOGAT cycle but also participates in the tricarboxylic acid (TCA) cycle, it constitutes a branching point between carbon (C) and N metabolism, and is subject to fluctuations according to the cellular N status. Hence, 2OG is an indicator of C as well as N availability and its level accumulates in response to N limitation [35]. Moreover, because glutamine is the product of GS it is also suitable to reflect the external availability of N to the cell in addition to 2OG [35].

A tight connection of the P_II_ and GS nucleotidylation cycles is ensured by the modification state of P_II_: adenylylation, and thus inactivation of GS by ATase strictly depends on the binding of unmodified P_II_, which accumulates during N-excess and high glutamine levels. On the other hand, deadenylylation and thus activation of GS by ATase only occurs upon binding of covalently modified P_II_(UMP), which accumulates during N limitation promoted by 2OG [25,29,36]. Hence, this bicyclic regulatory mechanism of enterobacteria constitutes a reciprocal control of the ATase- and UTase-catalyzed reactions by indicators of the cellular N status, 2OG and glutamine, to fine-tune GS activity accordingly (Figure 2) [28,37].

A third regulatory entity of enterobacterial GS is the widespread Ntr two-component system comprising the sensory histidine kinase NtrB and its response regulator NtrC. This system controls the N-dependent expression of the *glnA* gene, which encodes the GS monomers (Figure 2). The ATP-dependent autophosphorylation of NtrB yields active NtrC by transmission of the phosphoryl moiety. Subsequently, active NtrC promotes transcription of *glnA*, as well as other N-assimilatory genes, which also feature σ^54^-dependent promoters [40]. This process is antagonized by the emergence of unmodified P_II_ protein, which triggers dephosphorylation and thus inactivation of NtrC (reviewed by [41]). In summary, UTase probes the cellular N status via both glutamine and 2OG concentrations thereby influencing the levels of unmodified and modified P_II_ protein. This modification sensitizes or desensitizes GS for feedback inhibition by adenylylation/deadenylylation of the enzyme while the NtrB-NtrC system governs transcriptional control and therefore GS synthesis with respect to the cellular C/N balance (for a review see [39]).

GS regulation in enterobacteria appears relatively uniform, though investigation of an increasing number of bacteria beyond the enterobacterial family revealed striking differences in GS regulation among virtually every family investigated during the last two decades. Nevertheless, the assimilatory enzymes and several regulatory proteins involved appear to be mostly conserved in prokaryotes. For instance, the bifunctional GS of low G+C Gram-positive bacteria like *Bacillus subtilis*, which functions as both enzyme and regulator, is subject to feedback inhibition but devoid of posttranscriptional control [42]. During N-rich conditions, GS directly interacts with the transcriptional regulator of N assimilatory genes, TrnA and GlnK, an N-sensing P_II_-like protein. TrnA binding to GS triggers partial inhibition of enzymatic activity and ameliorates the impact of feedback inhibitors, especially of glutamine. In addition, GS binding obstructs DNA binding of TrnA. Under N-limiting conditions, GlnK forestalls interaction of TrnA with active GS, rendering the enzyme fully active [42,43]. In *Clostridium saccharobutylicum* NCP262, an endospore-forming obligate anaerobe, regulation of ammonium assimilation underlies a yet not fully understood mechanism encompassing post-transcriptional regulation of core enzymes via antisense RNAs as well as a specific RNA-binding regulatory protein of the ANTAR family, termed NitR [44], which is capable of transcription antitermination and thus induces expression of GS/GOGAT cycle genes upon N limitation [45]. Another mechanism of GS regulation was observed in several archaea where protein-protein interaction of GS with the P_II_-like protein GlnK was shown to increase GS activity in the presence of 2OG, while low 2OG levels thereof triggered GS inhibition by GlnK [46,47]. Taken together, these examples illustrate the complex and unique regulatory mechanisms of GS in diverse prokaryotes. This review aims to complement the body of knowledge on the diversified GS regulation throughout the prokaryotic domain by summarizing the insights gained into the exceptional mechanisms underlying GS regulation in cyanobacteria.

## 2. Cyanobacteria Evolved Exceptional GS Regulatory Mechanisms

As the evolutionary ancestors of chloroplasts, cyanobacteria represent a very ancient lineage and are the only bacterial group capable of performing water-splitting, oxygenic photosynthesis by combining the reductive power of two photosystems. This phylum shows substantial genomic and morphological divergence with unicellular and multicellular lifestyles, the latter showing a high degree of organization and differentiation. Cyanobacteria can be encountered in nearly any light-exposed habitat ranging from arctic regions, desert soils, rock surfaces, hot wells, and hypersaline lakes to fresh-water ecosystems, as well as ultraoligotrophic marine realms [48,49,50,51,52] and even non-light exposed habitats such as the deep terrestrial biosphere [53]. Cyanobacteria are utterly important in ecological terms and fundamentally affect global macronutrient cycles, as marine *Prochlorococcus* strains, for instance, account for 25% of the ocean’s net primary production [54]. Considering their metabolic performances and sheer abundance, cyanobacteria are prone to deplete nutrients in their vicinity, which renders nutrient deficiency a common factor for growth retardation. This is exemplified by the photosynthetic performance of early cyanobacteria, which caused the release of vast amounts of the photosynthetic byproduct O_2_ and simultaneous depletion of the majority of atmospheric carbon dioxide, which fundamentally affected living conditions on earth and paved the way for the evolution of aerobic metabolism and complex life [55].

For cyanobacteria, N represents a vital nutrient and accounts for up to 10% of their dry weight [56]. This is also illustrated by the fact that non-diazotrophic cyanobacteria, incapable of N_2_ fixation, tightly regulate their N assimilatory machinery and undergo substantial adaptations with respect to N availability [57]. For instance, prolonged N starvation eventually can result in degradation of the photosynthetic apparatus in a process designated as chlorosis [58]. Cyanobacteria are capable of assimilating dissolved N in form of ammonium, nitrate, and nitrite, and some can utilize urea, cyanate as well as several amino acids [56]. Notwithstanding, ammonium is the N species, which is directly incorporated by the GS/GOGAT cycle while other combined N sources require reduction prior to incorporation. Therefore, ammonium is the favored, least energy-demanding N source for most cyanobacteria [57]. Shortly after the discovery of GS in enterobacteria [7], the occurrence of GS/GOGAT and GDH was confirmed in the clade of cyanobacteria [59,60,61] and subsequent ^13^N-labeling experiments unequivocally established GS/GOGAT as the main N assimilatory pathway [62,63,64]. Apparently, GDH plays a rather auxiliary role in cyanobacterial N assimilation and imparts selective advantages in late stages of growth [65].

The cyanobacterial GS shares substantial similarities with the GSI of other bacteria, illustrated by the fact that it is able to complement a *glnA*-deficient *E. coli* mutant [66]. Nevertheless, the systems, which sense the N status and control GS in cyanobacteria, lack several known features characteristic for other, well-investigated bacterial clades. Purification and characterization of the GS from different cyanobacteria revealed a similar structure and subunit composition but, most strikingly, lacked covalent modifications characteristic of the enterobacterial GS [67,68,69]. Moreover, when compared to the enzyme of *E*. *coli*, GS of various cyanobacteria showed lower K_m_ values and a higher sensitivity towards common GS inhibitors such as a variety of amino acids [68]. Furthermore, cyanobacterial GS features a marked specificity for ATP and is greatly inhibited by AMP. Thus, GS may respond more vigorously to differences in energy charge and adenine nucleotide availability, which are known to fluctuate in response to environmental alterations in cyanobacteria [69,70].

### 2.1. Sensing of the Cellular N Status and Transcriptional regulation of the glnA Gene in Cyanobacteria

In cyanobacteria, the expression of the *glnA* gene is regulated with respect to the nature and abundance of the N source. N excess, e.g., induced by ammonium supplementation, results in low *glnA* expression, while cells grown on nitrate as sole N source feature elevated *glnA* levels only exceeded by N-starved cells [71,72,73,74,75]. Remarkably, the thoroughly investigated Ntr two-component system, which controls transcription of *glnA* in a wide range of bacteria, is absent in cyanobacteria [41,66]. Instead, the transcription of genes involved in N uptake and assimilation is mainly controlled by the highly conserved global transcriptional regulator NtcA, a protein of the CRP (the cyclic AMP receptor protein, also known as catabolite repressor protein) regulator family [76,77]. NtcA is restricted to cyanobacteria and can act both as an activator or repressor, depending on the location of its binding site with respect to the transcriptional start site (TSS) of the regulated genes [57]. Under N limitation, NtcA mediates the simultaneous up-regulation of multiple genes for N uptake systems (e.g., *amt1* encoding an ammonium transporter) and other N-related genes including *glnA* as well as *ntcA* itself [57,78,79,80].

**Figure 3:**
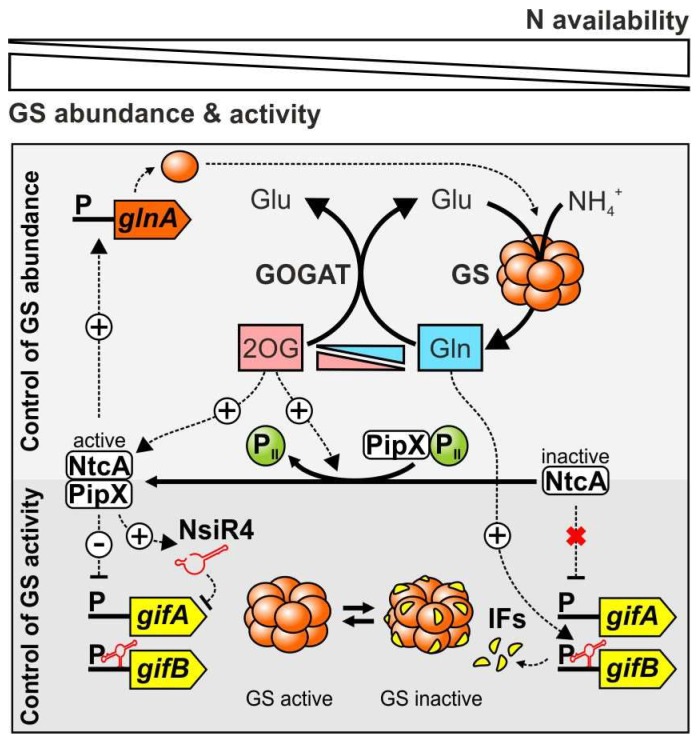
Regulatory network of GS in *Synechocystis* sp. PCC 6803. Increase in 2OG abundance during N deprivation promotes NtcA activation directly and via interaction with PipX. Active NtcA boosts transcription of the *glnA* gene encoding the GS monomers and of the sRNA NsiR4, which inhibits IF7 synthesis by interacting with the *gifA* mRNA. Simultaneously, transcription of the IF encoding *gif* genes is repressed by NtcA binding. N excess causes 2OG depletion, which results in PipX-P_II_ complex formation and inactivation of NtcA. Thus, IFs accumulate and inactivate GS in a concentration-dependent manner due to absent NtcA-mediated repression. Furthermore, N availability is also sensed by a glutamine riboswitch within the 5’UTR of *gifB*, which controls the synthesis of IF17 (*gifB*). IF: inactivating factor, P: promoter.

NtcA-dependent transcriptional regulation includes the interplay of the ubiquitous signal transduction protein P_II_ with the co-activator protein PipX, which modulate NtcA activity with respect to the cellular N status [81,82,83]. The regulatory circuit focusing on the *glnA* gene is summarized in Figure 3. Despite being crucial for N sensing in other bacterial groups, preliminary findings suggested that cyanobacteria do not perceive the cellular N status by probing glutamine levels [2]. Instead, 2OG was shown to be the metabolite, which governs NtcA activity and thus sensing of the N status in cyanobacteria [84]. As an indicator of cellular C/N balance, 2OG directly modulates the binding affinity of the homodimeric NtcA to its target promoters [84,85,86], which contain the NtcA recognition sequence GTA-N_8_-TAC [87,88,89]. Moreover, the small co-activator PipX is capable of coalescing in alternate complexes with either NtcA or the P_II_ protein [81]. Emergence of respective complexes is mutually exclusive, as high 2OG levels caused by N deprivation result in PipX-NtcA complex formation and up-regulation of N assimilatory genes including *glnA* (Figure 3) while PipX-P_II_ complex formation is prevented. On the other hand, low 2OG levels during N excess initializes PipX-P_II_ complex formation, which prevents PipX from boosting NtcA-mediated transcriptional regulation and significantly lowers NtcA binding affinities under these conditions [81,90]. Thus, this mechanism provides a functional connection of P_II_-mediated N signaling and NtcA-dependent gene regulation, which is unique to cyanobacteria and ensures proper expression of *glnA* and other N-related genes with respect to metabolic demands and N supply (Figure 3). Nevertheless, it should be noted that the interaction of PipX with P_II_ is not only tuned by changes in 2OG levels, but also by changes in ADP. ADP strongly enhances the affinity of P_II_ for PipX, and thus, increasing ADP levels allow P_II_-PipX complex formation even in the presence of 2OG [91].

### 2.2. Cyanobacterial GS Is Inactivated by Interaction with Small Proteins

In the early 1990s, GS in cyanobacteria was shown to be subject to a pronounced short-term inactivation upon addition of ammonium to nitrate grown cells [92]. Nevertheless, in contrast to the sophisticated adenylylation/deadenylylation system of enterobacteria, cyanobacterial GS was neither found adenylylated nor otherwise covalently modified [66]. Shortly after, it was shown that cyanobacterial GS, inactivated by ammonium supplementation in vivo, was reactivated in vitro by increasing pH, ionic strength or treatment with phosphatases, raising the possibility of non-covalent binding of any compound as the cause of GS inactivation [93]. Subsequent cross-linking experiments and mobility shift essays endorsed conclusions that rather polypeptides and not metabolites are involved in the observed inactivation [94]. Eventually, two homologous small proteins, namely the GS inactivating factors 7 and 17 (IF7 and IF17, respectively) were discovered due to the fact that two polypeptides of 7 and 17 kDa co-purified with ammonium-inactivated GS of the model strain *Synechocystis* sp. PCC 6803 (hereafter referred to as *Synechocystis*) [95]. These two proteins are encoded by the two separate genes *gifA* and *gifB* (glutamine synthetase inactivating factor A and B) with IF7 bearing remarkable resemblance to the C-terminal moiety of IF17 [95]. The regulatory interplay between GS and its IFs is illustrated in Figure 3.

In vitro analysis of heterologically expressed IF7, IF17, and GS of *Synechocystis* unequivocally demonstrated that both factors act independently from each other and do not require additional modifications to exert a concentration-dependent inactivation of GS [95]. Remarkably, in contrast to the enterobacterial adenylylation system, GS inhibition by small proteins in cyanobacteria renders the enzyme entirely inactive [95]. Generation of *ΔgifA* and *ΔgifB* single as well as a *ΔgifAgifB* double knockout strains rendered GS partially or fully active when grown with ammonium, demonstrating that the proteins encoded by these genes are exclusively responsible for GS inactivation and thus constitute a major layer of GS regulation in cyanobacteria [95].

### 2.3. The Biochemical Mechanism of GS Inactivation by Protein-Protein Interaction

In light of the reactivation of ammonium-inactivated GS by increasing pH values [93] and the high amount of positively charged residues in both IFs supported the idea of an electrostatic interaction with GS, likely on the same, negatively charged site of the enzyme [95]. In fact, three conserved arginine residues were found to be essential and equally important for the interaction of the *Synechocystis* IF7 with GS both in vivo and in vitro [96]. Substitution of one of these residues by either neutral (alanine) or negatively charged residues (glutamic acid) eradicated any interaction of IF7 with GS and rendered the enzyme fully active, thus indicating that loss of one junction between both proteins impedes any interaction [96]. In contrast, IF17 also features three conserved R residues, however substitution of these had a less pronounced effect on GS inhibition. Intriguingly, the importance of these three residues for IF17 function appears to be disparate as, for instance, substitution of R110, which is situated in the C-terminal moiety of IF17, the homologous region to IF7, had the most significant effect on protein function, while only exchange of all three R residues featured a fully active GS [96].

The stability of both GS inactivating factors is promoted by the interaction with GS in vivo [97]. Nevertheless, the IFs from *Synechocystis* vary in several aspects. IF17 is subject to proteolytic degradation stimulated by the absence of ammonium [97]. In contrast, stability of IF7 is not affected by N availability. Nevertheless, the IF7 protein appears to be degraded by the constitutively expressed Prp1/Prp2 metalloprotease, while the contribution of further proteases was suggested [97]. Interestingly, rapid IF17 degradation, which occurs in vivo upon ammonium removal, could not be observed in vitro; thus, additional factors must govern the N-dependent degradation of IF17, independent of *gifB* transcription. The mechanisms involved in the different IF17 degradation pattern, however, were not identified but may include the 82-residue N-terminal moiety, which is not present in IF7 [95,97]. This N-terminal part clearly contributes to protein stability: IF17 devoid of its N-terminus was outright susceptible to degradation while addition of the IF17 N-terminus to IF7 conveyed enhanced stability of that protein [96]. Even though the interaction of both IFs with GS mainly involves the same amino acids, IF17 has a stronger inhibitory effect on GS compared to IF7 [95]. This is explained by two distinct IF17 properties: the greater binding affinity to GS, which is associated with a lysine residue (K102) that contributes to the function of IF17 as well as its N-terminal region, which seems to improve GS inactivation [96]. 

Recently, analysis of chimeric enzymes of *Synechocystis* and the filamentous, dinitrogen-fixing strain *Anabaena* sp. PCC 7120 (hereafter referred to as *Anabaena*) suggested a possible coevolution of cyanobacterial GS and their corresponding IFs [98]. Each enzyme features two residues in the C-terminal moiety, which are crucial for specific interaction with their respective inactivating factors. Intriguingly, no conservation of these critical residues in the C-termini of other cyanobacterial GSs was observed, irrespective of the number of IFs possessed by these cyanobacteria, thus strongly suggesting a coevolution of GSs and IFs [98]. Furthermore, investigation of the GS-IF complex highlighted several possible mechanisms for protein-mediated GS inactivation [98]. Complex formation could cause alteration of the GS quarternary structure, which would result in perturbation of the active site and thus elimination of enzymatic activity. Another possibility is the contribution of enzymatically relevant residues in the interaction with IFs. Last, peripheral binding of IFs alongside the GS surface could impede the passage of substrates or products to or from the active site [98].

### 2.4. GS Inactivating Factors Are Common in Cyanobacteria and Transcriptionally Regulated by NtcA

Transcriptional analyses in *Synechocystis* revealed that expression of *gifA* and *gifB* was strongly increased upon ammonium addition while diminished expression of both genes was observed under N deficiency [99]. Therefore, *gifA* and *gifB* transcript accumulation is in accordance with the observed inactivation of GS in the presence of ammonium. Consequently, sequence analysis of the upstream regions of the *gifA* and *gifB* genes revealed potential binding sites for the global N transcriptional regulator NtcA, which center at the core promoter elements at −30.5 and −7.5 bp, respectively, thereby potentially mediating repression of the downstream genes. This assumption was confirmed as a non-segregated NtcA mutant featured constitutive expression of *gifA* and *gifB*, independent from the N source [99]. Upon N-limiting conditions, *gifA* and *gifB* transcription is repressed by NtcA binding and therefore inversely regulated to the GS structural gene *glnA*, which is activated by NtcA simultaneously [99]. Thus, NtcA and the GS IFs operate on transcriptional and posttranslational levels to adjust GS activity according to N availability (Figure 3).

Derived from the IF7/IF17 system of *Synechocystis* in which GS inactivating factors were discovered first, cyanobacterial GS inactivating factors can be subsumed into IF7-like and IF17-like classes with the exception of genome-streamlined marine picocyanobacteria like *Prochlorococcus*, which appear to lack full-length homologs [96]. Most cyanobacterial strains bear IF7-like inactivating factors [100] but other configurations can occur. For instance, *Thermosynechococcus elongatus* features two proteins homologous to IF17 while *Anabaena* harbors only one IF7-like inactivating factor (IF7A). Functional characterization of the IF7/*gifA* homolog of *Anabaena* confirmed that GS is also subject to IF-mediated inhibition in this cyanobacterium [101]. Nevertheless, the regulatory characteristics differ in several aspects from the system described in *Synechocystis*. Expression of the IF7A-encoding *gifA* gene is also regulated by the global N transcriptional regulator NtcA in *Anabaena* but in contrast to *gif* promoters in S*ynechocystis*, the upstream region harbors two NtcA binding sites. One site is centered at the −28.5 position, indicating a repressive role of NtcA for this promoter as the binding site features the very same distance to the −10 box in the NtcA repressed *gifA* promoter of *Synechocystis* [101]. Interestingly, the other site centers at −77.5 with respect to the TSS [101]. Despite the fact that binding motifs in canonical NtcA-activated promoters center at the −40.5 position, NtcA binding to the second site centering at position −77.5 is thought to activate *gifA* gene expression as several NtcA-activated promoters were shown to feature motifs upstream of −41.5 [99,100]. The simultaneous activation and repression of *gifA* expression upon NtcA binding in *Anabaena* is conflicting and the biological meaning is not understood. Nevertheless, *gifA* expression in *Anabaena* is strongly increased upon addition of NH_4_^+^, similar to the situation in *Synechocystis* [101]. In addition, another interesting finding of the latter study was the co-occurrence of a point mutation within the *gifA* gene in the *ntcA* mutant strain CSE2. This mutation leads to a premature stop codon, preventing synthesis of IF7A. Complementation of a WT *gifA* gene in the *ntcA* mutant strain resulted in poor growth, which points towards IF7A toxicity if *gifA* is not repressed by NtcA und thus de-regulated. Hence, the existence of two independent IFs in *Synechocystis* might explain why there is no fully segregated *ntcA* knockout mutant available for that strain thus far because single mutations, e.g., in *gifA* would not compensate the steady inactivation of GS.

In general, it can be stated that inactivation of GS is more efficient in strains, which possess two IF homologs. In *Synechocystis* for instance, the joint action of IF7 and IF17 during N-rich conditions rapidly inhibits GS activity and thereby, quickly restore the intracellular 2OG levels, which leads to repression of the *gif* genes once again. On the contrary, in strains harboring solitary IFs such as *Anabaena* this process is less effective, thus a rather continuous expression of *gif* genes is required to maintain appropriate levels of GS activity [95,101].

### 2.5. A Small Regulatory RNA Interacts with the gifA mRNA and Interferes with IF7 Production

In addition to transcriptional regulators, bacteria possess numerous and diverse means of RNA-mediated gene regulation. These regulatory RNA elements do not encode proteins and thus are non-coding. A major group of those non-coding RNAs (ncRNAs) can activate or repress gene expression at the post-transcriptional level by complementary base pairing with mRNAs. Those small regulatory RNAs (sRNAs) show short, imperfect base pairing interactions but frequently overlap with sequences required for translation initiation and hence, contribute to the specific and customized synthesis of the respective proteins [101,102,103,104].

In recent years, biocomputational predictions and studies mapping TSSs on a genome-wide scale gave rise to a plethora of sRNAs in cyanobacteria [89,105,106,107,108]. The expression of several sRNAs was vigorously enhanced upon N-limiting conditions indicating that they may act as regulatory elements in cyanobacterial N metabolism. For instance, three sRNAs upregulated during N depletion were identified in *Anabaena* and named N stress-induced RNAs (NsiR1, NsiR2, and NsiR3). Nevertheless, their detailed functions are yet to be elucidated [89,109,110]. In *Synechocystis*, the sRNA NsiR4 was verified as one of the most strongly induced transcripts during N-limiting conditions [106]. Albeit absent in α-cyanobacteria, NsiR4 is conserved among distantly related cyanobacteria as homologs were found in at least 38 cyanobacterial genomes covering all five morphological subsections [111]. These findings provided a strong hint that NsiR4 indeed possesses a biological function. Interestingly, examination of genomes and transcriptomes of different cyanobacteria including *Anabaena* [89], *Synechoccoccus* sp. PCC 7002 [112] and *Synechocystis* sp. PCC 6714 [113] revealed the existence of two distinct NsiR4 forms, which differ in length and appear to be mutually exclusive as no co-occurrence could be observed. Nevertheless, in all cases a perfect NtcA binding motif was found upstream of NsiR4 homologs centering around −41.5 bp with respect to the TSS. Accordingly, NsiR4 expression is activated upon N depletion, which was experimentally verified in *Synechocystis* and *Anabaena* [111].

Intriguingly, the *gifA* gene was computationally predicted as the most promising NsiR4 target and the direct interaction of both RNAs was experimentally verified [111]. Consistent with previously examined sRNAs, which diminish target stability upon binding [114] knockout of NsiR4 led to an increased *gifA* mRNA abundance while NsiR4 overexpression decreased *gifA* transcript abundance compared to the WT, i.e., after adding ammonium. Subsequent analysis clearly demonstrated that the interaction of NsiR4 with the *gifA* transcript negatively affects IF7 protein synthesis and in turn also impacts GS activity [111]. The transcriptional regulation of *gifA* by NtcA is obviously not sufficient to control GS activity and hence, an additional post-transcriptional mechanism has evolved that helps to determine whether or not IF7 is produced (Figure 3). With this, NsiR4 was the first sRNA shown to regulate the assimilation of a macronutrient in bacteria directly as it is involved in controlling the entry of inorganic N into cellular metabolism. Very recently, also a nitrogen stress repressed RNA (NsrR1) has been identified in heterocyst-forming cyanobacteria which regulates, in a coherent feed-forward loop together with NtcA, a protein involved in phycobilisome degradation under N limitation [115]. Nevertheless, RNA-based N regulation is not restricted to cyanobacteria as a diverse range of prokaryotes meanwhile was shown to possess sRNAs that control N metabolism (reviewed by [116]).

### 2.6. A Glutamine Riboswitch in the gifB mRNA Regulates IF17 Synthesis

Yet another type of ncRNAs are riboswitches, which fulfill integral regulatory tasks in bacteria [117,118,119]. Riboswitches reside in the 5’UTR of mRNAs and can modulate the expression of downstream genes by ligand-induced conformational changes. Riboswitches are composed of an aptamer, which specifically binds the ligand (e.g., metabolites, inorganic ions), and an expression platform, which determines the read out of genetic information by affecting transcription, translation or RNA processing [120,121,122,123]. In 2010, a myriad of highly structured ncRNAs with potential biological functions were determined computationally by examining genome and metagenome databases [124]. Two classes of RNA motifs, related in both structure and sequence, were shown to exclusively occur in cyanobacterial genomes and marine metagenomes of environmental samples. Several of these structured RNAs were found upstream of genes encoding for proteins pivotal for N regulation, such as the N regulatory protein PII, GS, and ammonium transporters, thus suggesting a potential regulatory role in N metabolism [124]. These conserved sequence elements were termed *glnA* and downstream-peptide (DP) motifs and annotated as RF01739 and RF01704 in the Rfam database [125], respectively. Intriguingly, both the *glnA* motif of *Synechococcus elongatus* as well as the DP motif of *Synechococcus* sp. CC9902 were shown to alter their secondary structure upon selective binding of l-glutamine in vitro, which classified them as glutamine-binding aptamers [126]. For the first time, this study acknowledged the possibility that glutamine might be also involved in perceiving the N status in cyanobacteria. Cyanobacteria were believed to lack glutamine signaling because homologs of the known, glutamine sensing enzymes were not found in cyanobacterial genomes [2].

Recently, detailed analysis revealed a widespread occurrence of the glnA aptamer in cyanobacterial strains. The DP aptamer, however, is apparently restricted to the monophyletic group of marine picocyanobacteria where it frequently exists in multiple copies [127]. Within the available cyanobacterial genome sequences the aptamers were mainly found to be associated with genes encoding for proteins, which harbor the domain of unknown function 4278 (DUF4278). Other associations such as with genes for GS, P_II_, and ammonium transporters as reported previously [124] appear to be restricted to sequences of environmental samples and hence yet uncultured strains. Interestingly, DUF4278 covers the N-terminal moiety of the GS inactivating factor IF17 encoded by gifB. This suggested that IF17 synthesis might be also controlled by a ncRNA, namely by a glutamine riboswitch. In *Synechocystis* it was experimentally proven that the glutamine binding aptamer present in the 5’UTR of *gifB* undergoes structural modulations, which in turn impact the production of IF17 [127]. These results unequivocally demonstrated that the described *glnA* aptamer represents the metabolite-sensing domain of a glutamine-binding riboswitch, thus renaming to type 1 glutamine riboswitch was proposed. Investigation of a DP aptamer of a *Prochlorococcus* strain revealed that these elements are also part of functional glutamine riboswitches and activate gene expression upon glutamine binding similar to the type 1 glutamine riboswitch. Hence, for those containing the DP aptamer the designation type 2 glutamine riboswitch was suggested [127].

The glutamine riboswitches are restricted but common to cyanobacteria and their discovery added an important piece in the puzzle of glutamine synthetase regulation in this bacterial group (Figure 3). Previously, 2OG-dependent transcriptional regulation of *gifA* and *gifB* by NtcA was considered to be exclusively responsible for the control of GS activity in cyanobacteria [84,99]. Nevertheless, the riboswitches were proven to be required for sufficient GS regulation as well and are thus additional key elements that act in a glutamine-dependent manner [127]. Hence, cyanobacteria also use glutamine as a signaling molecule for perceiving the N status, however via a unique RNA-based sensing mechanism.

### 2.7. The Regulation of GS in Marine Picocyanobacteria is Unclear

Marine picocyanobacteria constitute a monophyletic group within the cyanobacterial realm, which displays several remarkable traits. One representative that attained the reputation of a model organism for marine ecology is the genus *Prochlorococcus* [128]. *Prochlorococcus* diverge in genetically distinct ecotypes along the water column, mainly caused by the aptitude to cope with different light intensities resulting in a tailor-made outfit of photosynthetic pigmentation for the given light conditions [129]. While those strains possess the smallest genomes and cell sizes of any photosynthetic organism [130,131] it is the most abundant organism in the ocean and supposedly on earth thus being of fundamental ecological importance for global nutrient cycles [54]. The extremely nutrient-deficient niches occupied by these organisms prompted several outstanding adaptions: vast genome streamlining [132,133] accompanied by the substitution of protein regulators with ncRNAs [134] and a low GC content [132]. N limitation imposes rigorous constraints on the boundaries of biomass accumulation and primary productivity in marine ultraoligotrophic habitats [135]. Thus, N cost-minimizing measures such as the preference for amino acids reduced in N content [136] and the augmented usage of internal TSSs upon N limitation, resulting in shortened proteins with abated N content [137] allow *Prochlorococcus* strains to flourish in N-poor environments of the open ocean.

Despite the cost-minimizing loss of proteinaceous regulators, these strains retained NtcA and P_II_, the key proteins for N sensing and regulation of N assimilation in cyanobacteria [138]. Albeit featuring a similar structure, subunit composition, physicochemical properties, and enzymatic performance compared to the GS of other cyanobacteria, early studies in *Prochlorococcus* illustrated that N shortage causes a different response compared to other cyanobacteria [139,140]. For instance, GS activity in *Prochlorococcus* MED4 was shown to be substantially enhanced during long-term N starvation, while GS protein levels remained relatively constant under these conditions [140] thus contrasting common regulatory traits of other cyanobacterial GSs in which N deprivation causes a significant increase in GS protein abundance and activity [141].

The steady GS abundance in response to N availability probably resulting from an inexpensive, less sophisticated regulation of this pivotal enzyme may impart evolutionary advantages in habitats occupied by *Prochlorococcus*. These display rather stable N supplies rendering excessive GS regulation superfluous and costly [142]. Nevertheless, the *glnA* gene was shown to be one of the top responding transcripts during N stress in *Prochlorococcus* and appears to be regulated by NtcA, consistent with observations made in other cyanobacteria [143]. These contradicting results, substantial alterations on the transcriptional level concomitant with steady protein abundances may be due to differential GS regulatory mechanisms on the posttranscriptional level in observed strains. Since the apparent protein level results from the antagonistic processes of protein synthesis and degradation, constant GS protein levels during N-starvation may depend on a degradation process triggered by N deprivation. An explanation could be provided by the susceptibility of the *Prochlorococcus* GS for metal-catalyzed oxidation (MCO) prior to degradation upon N limitation [144,145], which also occurs in *E. coli* and *B. subtilis* [146,147]. This process promotes the degradation of unessential proteins to provide amino acids for the synthesis of proteins crucial to cope with nutrient limitation thus meeting the challenges imposed to *Prochlorococcus* strains.

Most compelling, the previously described type 2 glutamine riboswitch, which always occurs in multiple copy numbers and appears restricted to marine picocyanobacteria, is functionally associated to genes encoding for DUF4278 containing proteins. Intriguingly, these proteins share substantial similarities with the N-terminus of IF17 [127], which was shown to be disposable for IF17 function but substantially promotes protein stability in *Synechocystis* [96]. Previous findings ruled out IF-mediated GS regulation in the clade of genome-streamlined marine picocyanobacteria living in ultraoligotrophic habitats that feature a monotonic nutritional nourishment [96,142]. Nevertheless it cannot be excluded that the GS and corresponding IFs of picocyanobacteria underwent co-evolutionary adaptions, which enables the interaction with these putative inhibitory proteins. This is supported by the finding that GS/IF-interaction is somewhat specific since GS of *Anabaena* could not be inhibited by IF7 and IF17 of *Synechocystis* in vitro [101]. For instance, the gene *PMM1846* of *Prochlorococcus* strain MED4 (also referred to as CCMP1986/NIES-2087) encodes a small protein of 7 kDa that exhibits striking resemblance to IFs of other cyanobacteria. It bears DUF4278, homologous to the N-terminus of IF17, and harbors a glutamine riboswitch in its 5’UTR that boosts gene expression during N excess [127]. Moreover, it appears to be transcriptionally regulated with respect to N availability, as a NtcA consensus motif [87] is located in its promoter region. Finally, the protein encoded by *PMM1846* shares several physicochemical properties with IFs, which are characteristic for proteins that partake in electrostatic protein-protein interactions such as the prevalence of “electrostatic targeting residues” like arginine [96]. Nevertheless, extensive analysis is required to clarify whether these genes indeed encode IF-like inhibitors of GS in the inherently underinvestigated clade of picocyanobacteria.

### 2.8. GlnN Represents Another GS in Cyanobacteria

As mentioned, GS splits in three distinct classes, which significantly differ in subunit size, subunit arrangement and sequence. First assumptions that another glutamine synthesizing enzyme exists in cyanobacteria were furnished by a *glnA*-deficient mutant of *Synechococcus* sp. PCC 7002, which was able to grow without glutamine and retained most of the wild-type glutamine biosynthetic activity [75]. Shortly afterwards, the generation of a *Synechocystis glnA* mutant strain, which exhibited WT like growth in nitrate-containing media, devoid of glutamine supplementation strongly supported the idea of an additional GS enzyme, which facilitates N assimilation during *glnA* deficiency [12]. Subsequent complementation of a *glnA* deficient *E. coli* mutant strain with a *Synechocystis* library led to the discovery of the *glnN* gene encoding a GS enzyme, which only shares a minor resemblance with the amply investigated GSI and GSII mostly restricted to 17 strongly conserved residues in the predicted ATP and cation binding sites. However, the protein was found homologous to the GSIII, which was previously described in the family of *Bacteroidaceae* with a sequence-derived subunit size of 75 kDa and a putative hexameric arrangement [12,13,148,149]. Interestingly, *glnN* expression is substantially affected by the N source as ammonium supplementation suppresses *glnN* expression while N-free media strongly induced *glnN* transcript accumulation. Being dispensable for N assimilation and WT-like growth rates, GSIII accounts for 3% of total GS activity in nitrate-grown *Synechocysti*s cells [12]. In contrast, total GS activity is raised upon N-limiting conditions during which GSIII provides about one fifth of total glutamine biosynthetic activity, as it was shown by a comparison of WT and *glnN*-deficient strains [12]. Moreover, the conjecture that a *Synechocystis glnA*/*glnN* double mutant was not viable illustrates the fact that there are most likely no other GS enzymes present in this strain [12]. Subsequent purification and characterization of the GSIII protein confirmed the previously predicted hexameric subunit stoichiometry and molecular mass of ~79 kDa [12,150]. Furthermore, it was shown that GSIII features similar kinetic properties and requires the same substrates and cofactors as described for the cyanobacterial GSI [67,150]. Considering that transition-state analogs of γ-glutamylphosphate, which irreversibly inhibit GSI, had the same effect on GlnN activity, while catalytically crucial residues are conserved in both enzymes, strongly suggests that the catalytic mechanism of GSIII matches the situation in GSI [150]. Furthermore, utilizing GSIII-specific antibodies, it was shown that several other cyanobacterial strains possess the *glnN* gene whose expression is induced upon N starvation, consistent with observations made in *Synechocystis*, while diazotrophic cyanobacteria apparently lack the *glnN* gene [150]. Even though the *glnN* promoter sequence features an imperfect NtcA consensus motif as well as N-dependent expression patterns, first attempts to show *E. coli*-expressed NtcA binding failed and it was assumed that interaction requires additional factors [76]. Subsequent scrutiny of NtcA-deficient strains, however, unequivocally demonstrated that *glnN* is another NtcA regulated gene in *Synechocystis* [58,99]. In light of these data it is tempting to speculate whether GSIII is particularly important for prolonged N starvation upon which non-diazotrophic cyanobacteria pull out all the stops to scavenge every available ammonium molecule. Notably, in *Synechococcus* sp. PCC 7942 GSIII was shown to promote the synthesis of phycobiliproteins when chlorotic cells were supplied with limiting amounts of combined N [58]. Hence, GSIII enhances the incorporation of combined N to recover after long-term N starvation as phycobiliproteins are utilized as N reservoirs for ensuing phases of N starvation. It remains enigmatic whether GSIII in *Synechocystis* and other GSI bearing strains is regulated by IFs. Given that GSIII in *Synechocystis* only occurs upon prolonged N starvation in physiologically relevant levels, a circumstance that causes fully active enzymes [12], IF-dependent inhibition of GSIII would be rather redundant. Recently, physiological examination of GSIII from *Synechococcus* sp. WH7803 revealed an unusual regulation of this enzyme as GSIII activity in this strain is highly responsive to light-dark changes while being unaffected by N availability [151]. Thus, the assumption that GSIII promotes recovery after prolonged N starvation [58] appears unlikely in *Synechococcus* sp. WH7803. Given that all sequenced *Prochlorococcus* strains lack the *glnN* gene, it appears that GSIII was lost in the course of streamlining in environments with stable nutrient availability [151]. Therefore, *Synechococcus* sp. WH7803 could be on the verge of losing its GSIII and the loss of its regulation by N availability might be the first step in this process.

Intriguingly, one of the previously discovered *glnN-*bearing strains, *Pseudanabaena* 6903 was shown to lack a *glnA* gene and thus was the first known cyanobacterium in which only GSIII accounts for the biosynthesis of glutamine [152]. More recently, analysis of *Synechococcus* sp. RS9917 revealed that this marine cyanobacterium possesses two GSIII enzymes and lacks GSI [151]. In contrast to the situation in *Synechocystis* where nitrate-supplementation causes basal GSIII levels, *glnN* expression is strongly induced in nitrate-grown as well as N-starved cells of *Pseudanabaena* 6903, while ammonium addition triggers a rigorous inhibition of enzymatic activity accompanied by steady protein levels. This was not observed when N-starved cells were subjected to nitrate [152]. These findings clearly illustrate that GSIII in *Pseudanabaena* 6903 is subject to a yet unknown, rapid, ammonium-dependent inactivating mechanism, as minor GS activity would already be sufficient to ensure proper N supply under these conditions. Interestingly, *glnN* transcription in *Pseudanabaena* 6903 was also shown to be controlled by N, which is supported by the fact that the *glnN* promoter harbors an imperfect NtcA consensus sequence which is bound by NtcA of *Synechocystis* [152]. Thus, the regulatory pattern of *glnN* expression in *Pseudanabaena* 6903 is similar to the N-dependent control of *glnA* expression in *Synechocystis* as NtcA strongly induces the transcription of N-related genes during growth on nitrate or N-depleted media [76]. Thus, by employing NtcA-mediated N control of *glnN* transcription, *Pseudanabaena* 6903 deploys sufficient levels of its sole GS enzyme to meet metabolic demands during N-limiting conditions [152]. Unfortunately, there are no publicly available genomes of *Pseudanabaena* 6903, and thus the question whether GSIII is subject to protein-mediated regulation or an utterly different mechanism in this strain remains unanswered.

## 3. Concluding Remarks

Due to their photosynthetic lifestyle cyanobacteria are interesting hosts for the production of fuel components and valuable chemicals using light and CO_2_. However, their rising importance as microbial cell factories in biotechnology is contradicted by the current state of knowledge about metabolic regulation. As exemplified by the regulation of glutamine synthetase, cyanobacteria differ from other bacterial groups in many aspects. Hence, data that were obtained from genetic models like *E. coli* or *B. subtilis* can often not be applied. The intriguing example of cyanobacterial GS regulation suggests that further metabolic reactions might be controlled differently compared to other bacteria and that many regulatory factors still await discovery and/or characterization. Altogether, further basic research is required to make cyanobacteria more definable for metabolic engineering and thus also amenable for biotechnological applications.
